# The Closing Days of Our College Courses

**Published:** 1895-03

**Authors:** 


THE closing days of many of our college courses are rapidly approaching, and while we feel sure that in all the schools with few exceptions, this year's course has been broader, better and more thorough in many directions than any of its predecessors, still in none has it attained the height and scope that every true friend of veterinary education wishes for. At no time of the year are those engaged in teaching better situated to judge of the deficiencies in the college course, and to note where the needs are greatest, the weak spots most dangerous; and it is always a good time to sum up the work accomplished, the good achieved at the close of the session. Every student coming before his preceptor will instruct the latter how well he has done his work, how clearly and completely he has covered the sphere of his teaching; how far short the time allotted for instruction has proven to the disadvantage of the student; and it behooves one and all to strengthen and enlarge his part of the work, for we have reached and passed the milestone where a fairly well-educated pupil will become an efficient and worthy veterinarian. There is no longer any room, even in towns and country districts, for half-qualified veterinary surgeons. The book worm or the sponge-like student, who absorbs his knowledge and lacks the training to make it useful and effective, but who is armed with sharp-edged instruments, and little appreciates their danger when improperly used, must give way to the trained student whose education has been obtained through a graded, systematic course of drilling and practical instruction.

